# Public opinions about supervised smoking facilities for crack cocaine and other stimulants

**DOI:** 10.1186/s13011-016-0052-7

**Published:** 2016-02-09

**Authors:** Carol Strike, Nooshin Khobzi Rotondi, Tara Marie Watson, Gillian Kolla, Ahmed M. Bayoumi

**Affiliations:** Dalla Lana School of Public Health, University of Toronto, 155 College Street, Toronto, Canada; Musculoskeletal Health and Outcomes Research, Li Ka Shing Knowledge Institute, St. Michael’s Hospital, 30 Bond Street, Toronto, Canada; Centre for Research on Innercity Health, Li Ka Shing Knowledge Institute, St. Michael’s Hospital, 30 Bond Street, Toronto, Canada; Department of Medicine, University of Toronto, 1 King’s College Circle, Toronto, Canada; Institute of Health Policy, Management, and Evaluation, University of Toronto, 155 College Street, Toronto, Canada; Division of General Internal Medicine, St. Michael’s Hospital, 30 Bond Street, Toronto, Canada

**Keywords:** Supervised smoking facilities, Public opinion, Telephone survey, General population, Adult, Canada

## Abstract

**Background:**

The purpose of this study was to estimate awareness and opinions about supervised smoking facilities (SSFs) for smoking crack cocaine and other stimulants and make comparisons with awareness and opinions about supervised injection facilities (SIFs) in Ontario, Canada.

**Methods:**

We used data from a 2009 telephone survey of a representative adult sample. The survey asked about awareness of, and level of support for, the implementation of SSFs and SIFs. Data were analysed using statistical models for complex survey data, which account for stratified sampling and incorporate sampling weights.

**Results:**

A total of 1035 participated in the survey. Significantly fewer had knowledge about SSFs (17.9 %) than about SIFs (57.6 %). Fewer strongly agreed with implementation of SSFs (19.6 %) than SIFs (28.3 %). Just over half (51.1 %) of participants somewhat agreed or disagreed, 15.7 % strongly agreed, and 10.6 % strongly disagreed with implementing both SSFs and SIFs.

**Conclusions:**

Members of the public in Ontario had little knowledge of SSFs compared to SIFs. Recent federal government changes in Canada may provide the leadership environment necessary to ensure that innovative, evidence-based harm reduction programs such as SSFs are developed and implemented.

## Background

The past thirty years have seen an expansion in public health programming to prevent drug-related harms for people who inject illicit drugs such as heroin, other opioids, cocaine and crack cocaine, and crystal methamphetamine. Well-known examples include needle and syringe programs and methadone maintenance treatment, both of which are effective at reducing human immunodeficiency virus (HIV) transmission [1, 2]. In recent years, there have been increasing calls to base drug policy and programming on evidence instead of ideology [3], including for example the implementation of supervised consumption facilities. These programs have been implemented in some countries to provide a hygienic environment where clients can inject and/or smoke illicitly obtained drugs [4].

Supervised injection facilities (SIFs) are a common type of consumption facility and a programmatic innovation with the goal of addressing injection-related harms and risks, including: reducing transmission of HIV, hepatitis C virus (HCV), and other blood-borne infections; decreasing morbidity, overdose and mortality associated with public drug use; minimising public disturbances and drug-related litter; and increasing contact between people who use drugs and health and social services [5–7]. Across the world, there are an estimated 90 supervised consumption facilities implemented where problem drug use continues despite the availability of drug treatment, needle and syringe programs, social services, and attempts by police to reduce drug-related public disorder and drug markets [8, 9]. A large and growing literature from Australia, Europe, and Canada has demonstrated numerous benefits of SIFs [10–16].

Across North America and Europe, smoking of stimulants such as crack cocaine is prevalent and associated with many negative health and social effects [17–21]. Reports suggest that the sharing of smoking equipment is common [22] and potentially exposes smokers to HCV and tuberculosis [23–26]. However, public health responses to these harms have been slower to develop than those related to injection drug use [22]. Nevertheless, some Canadian public health departments distribute low-cost or free safer crack cocaine smoking equipment (e.g., glass stems and mouthpieces) to reduce equipment sharing and potential injuries to the hands and mouth from using damaged smoking equipment [27]. In the United Kingdom, where smoking of heroin is more prevalent, some programs now distribute foil sheets designed to encourage the transition from injecting to heroin smoking [28]. Similar to SIFs, supervised smoking facilities (SSFs) operate in some European countries to reduce illicit drug smoking-related harms such as disease transmission, morbidity associated with public drug use, public disorder, drug-related litter, and limited access to health and social services [6].

There are no legally sanctioned SSFs in North America, although there have been calls for implementation of these programs [29, 30]. Results from an ethnographic study of an unsanctioned SSF in Vancouver, Canada operated by a drug user-led organisation showed the potential of SSFs to attract highly vulnerable people who smoke crack cocaine and to minimise client exposure to interpersonal violence [31]. However, the study showed only modest potential to reduce crack pipe sharing [31]. In other cities in Canada where crack cocaine smoking is prevalent, over two-thirds of people who smoke crack cocaine reported a willingness to use a SSF [5, 30].

Interest in these facilities has been raised by both public health officials and advocates in Canadian cities where crack cocaine smoking is prevalent [5, 32]. To our knowledge, there are no published studies that report public perception of SSFs or reports comparing opinions about SSFs relative to SIFs. However, public opinion is a factor in decision making regarding implementation of public health programs [33, 34]. The goal of our study was to estimate the level of public awareness of SSFs in general and relative to SIFs. In addition, we estimated the level of public agreement or lack thereof regarding implementation of SSFs, both in general and with respect to specific SSF goals.

## Methods

### Study design and data source

We analysed data from the Centre for Addiction and Mental Health (CAMH) Monitor survey, an annual cross-sectional survey that used computer-assisted telephone interviews to ask participants from Ontario, Canada about drug issues and policies as well as substance use and mental health. We used the 2009 survey, in which about 2000 adults were selected. Sampling followed a two-stage probability design. First, households were sampled using randomly selected telephone numbers within six regional strata. Second, the person within each household who was both age 18 or over and had the most recent birthday was selected. Fluency in English or French was required for eligibility. All participants provided informed consent. Additional information is available in the CAMH Monitor technical guide [35]. This study was approved by the Research Ethics Board at the Centre for Addiction and Mental Health.

For six of the months in 2009, survey participants were first asked about their opinions regarding SIFs and next asked about their opinions related to SSFs [36]. Prior to being asked for their opinions, participants were told that they would be asked questions about SIFs and were provided with the following description: “The Vancouver supervised injection facility, ‘Insite’, provides a place supervised by health care workers for drug users to inject their drugs. Several other cities in Canada are considering starting up similar programs.” Participants were asked if they strongly agreed, somewhat agreed, somewhat disagreed or strongly disagreed with each of the following statements:

1. Supervised injection facilities should be made available to injection drug users, to encourage safer drug injection.
2. Supervised injection facilities should be made available if it can be shown that they reduce overdose deaths or infectious disease among users.
3. Supervised injection facilities should be made available if they can increase drug users’ contact with health and social workers.
4. Supervised injection facilities should be made available if it can be shown that they reduce neighbourhood problems related to injection drug use.

After asking about SIFs, interviewers informed participants that, “Several cities in Canada are considering starting up similar facilities supervised by health care workers for drug users to smoke drugs like crack cocaine and methamphetamine. The next few questions are about your views on these facilities.” Specifically, participants were asked:

1. Have you ever read, seen or heard any information about supervised smoking facilities?
2. Please indicate if you strongly agree, somewhat agree, somewhat disagree or strongly disagree with the following statements:
  - Supervised smoking facilities should be made available to people who smoke drugs like crack cocaine and methamphetamine to encourage safer drug consumption
  - Supervised smoking facilities should be made available if it can be shown that they reduce infectious disease among to people who smoke drugs like crack cocaine and methamphetamine
  - Supervised smoking facilities should be made available if they can increase drug users’ contact with health and social workers
  - Supervised smoking facilities should be made available if it can be shown that they reduce neighbourhood problems related to consumption of drugs like crack cocaine and methamphetamine.

In contrast to Firestone-Cruz and colleagues [37], we created a composite measure of public opinion to highlight those with mixed opinions about SIFs. Focus group data from residents, business owners, and community service providers in our study also revealed high levels of ambivalence about SIF implementation [38]. We classified responses for each of the latter four questions into three categories: strongly disagree; somewhat agree or disagree (participants who might change their views); and strongly agree. Finally, we grouped responses to the four goals into a single composite variable using the following categories:

- Strongly agreed: participants who strongly agreed with all four goals for SSFs and SIFs
- Strongly disagreed: participants who strongly disagreed with all four goals for SSFs and SIFs
- Mixed opinions: all other patterns of responses

### Analysis

We used statistical models for complex survey data, which account for stratified sampling and incorporate sampling weights. These weights reflect study design and population characteristics such that the final survey results are representative of the Ontario population aged 18 years in the survey year. Sampling weights also provide accurate confidence intervals. A full description of the analytic methods can be found in a previously published study [36]. We compared the sample who answered questions to the entire population sample and to data from the 2006 Ontario Census. We performed chi-square tests of independence, taking into account the complex survey design, to determine the association between SSF awareness and each of the four specific SSF goals. We used a two-tailed p-value threshold of 0.05 to assess statistical significance and did not adjust for multiple comparisons.

We analysed independent predictors of knowledge of SSF using logistic regression and of support for SSF goals using multinomial logistic regression, with a 3-level dependent variable based on the composite support variable. Both methods used weights for complex survey designs. We used a non-parsimonious approach for each method and included all potential covariates listed in Table 1. Multinomial regression results are reported compared to the “mixed opinions” group as both the relative risk of strongly agreeing and of strongly disagreeing with the goals of SSFs.

**Table 1:** Demographic characteristics^a^

Characteristic	Weighted percent	Total survey	Census	Sample size; Design degrees of freedom	*p*-value; Chi-squared degrees of freedom
Female	55.0 %	51.5 %	51.9 %	1035; 1029	0.049; 1
Age (years)					
18 to 34	25.5 %	26.3 %	28.5 %	999; 993	0.012; 2
35 to 54	39.2 %	41.7 %	40.4 %		
55 and older	35.3 %	32.0 %	31.1 %		
Employment status					
Employed	63.2 %	63.7 %	64.5 %	1020; 1014	0.654; 2
Unemployed	4.5 %	5.1 %	4.1 %		
Other (student, retired, homemaker, disability, etc.)	32.3 %	31.1 %	31.4 %		
Household income in the past year before taxes					
Less than $30,000	13.0 %	11.4 %	12.6 %	730; 724	0.952; 3
Between $30,000 and $49,999,99	16.1 %	15.6 %	16.6 %		
Between $50,000 and $79,999,99	24.1 %	26.0 %	24.6 %		
More than $80,000	46.8 %	47.0 %	46.1 %		
Highest level of education attained					
Less than high school	10.5 %	10.6 %	18.6 %	1018; 1012	<0.001; 3
Completed high school	20.4 %	21.2 %	27.8 %		
Some post-secondary (college or university)	34.3 %	35.6 %	32.1 %		
University degree	34.8 %	32.7 %	21.4 %		
Marital Status					
Married/living with partner	71.0 %	69.1 %	62.9 %	1014; 1018	<0.001; 2
Previously married (divorced, widowed, separated)	10.6 %	10.6 %	14.1 %		
Never married	18.4 %	20.3 %	23.1 %		
Smoking Status			NA	1028; 1022	
Current	17.9 %	18.6 %			
Former	25.7 %	26.5 %			
Never	56.4 %	54.9 %			
Religious Service Attendance, past 12 months			NA	969; 963	
None	35.8 %	38.1 %			
1 t0 6 times	29.3 %	27.7 %			
7 or more times	34.9 %	34.2 %			
Alcohol use in the past 12 months	79.0 %	79.1 %	NA	1031; 1025	
Cannabis use in the past 12 months	11.8 %	13.2 %	NA	1033; 1027	
Fair or Poor Self-reported Health	9.6 %	10.5 %	NA	1029; 1023	
Fair or Poor Self-reported Mental Health	6.2 %	5.7 %	NA	1025; 1019	
Health care worker	6.8 %	6.1 %	NA	908; 902	
Immigrant to Canada	27.2 %	27.5 %	NA	1014; 1008	
Urban residence	80.3 %	82.9 %	NA	1035; 1029	

^a^Proportions estimated from one-way tables using complex survey designs. Total survey sample size = 2037. Census estimates are from the 2006 Canadian Census individual microuse data (http://www5.statcan.gc.ca/olc-cel/olc.action?ObjId=95M0028X&ObjType=2) for Ontario population aged 18 and older using individual weighting factors. *P*-values compare values in the current sample to population estimates using Pearson’s chi-square test. *NA* denotes not available

All analyses were completed using SAS 9.3 (SAS Institute), R 3.0.2 (R Core Team), and Stata version 13.1 (StataCorp).

## Results

In 2009, 1035 participants were asked questions about SIFs and SSFs. After applying survey weights, the typical respondent was female (55.0 %), aged 35 and over (72.2 %), employed full-time (62.4 %), and had completed at least some post-secondary education (68.1 %; Table 1). The participants who answered questions about SIFs and SSFs had a similar employment and income distribution as the Canadian population but women, older people, people who were married, and well-educated people were somewhat over-represented. At least one question regarding opinions about SIF or SSF implementation was not answered by 182 and 198 participants, respectively.

Using complex survey designs to estimate proportions, significantly fewer participants had ever read, seen or heard any information about SSFs (17.9 %, 95 % CI 15.1 % to 21.1 %) than SIFs (57.6 %, 95 % CI 53.9 % to 61.2 %); the difference was 39.7 % (95 % CI 35.8 % to 43.6 %; *p* < 0.001; difference estimated using the delta method using a t-distribution with 1010 degrees of freedom). Among those with no prior knowledge of SSFs, 48.8 % also had no prior knowledge of SIFs and among those with no prior knowledge of SIFs, 95.4 % also had no prior knowledge of SIFs thus 40.1 % of the population had no prior knowledge of either type of facility and 15.9 % had prior knowledge of both types (data not shown). Independent predictors of knowledge of SSFs included older age, male sex, having a university degree, and being a health care worker; people who immigrated to Canada were less likely have prior knowledge of SSFs (Table 2).

**Table 2:** Multvariable analysis of knowledge of supervised smoking facilities

Variable	Odds ratio (95 % Confidence Interval)
Age (per decade)	1.17 (1.02 to 1.34)
Male sex	1.88 (1.22 to 2.92)
Household income in the past year before taxes	
Less than $30,000	1.00 (Referent)
Between $30,000 and $49,999,99	0.63 (0.31 to 1.29)
Between $50,000 and $79,999,99	0.57 (0.28 to 1.16)
More than $80,000	0.91 (0.44 to 1.89)
Highest level of education attained	
Less than high school	1.00 (Referent)
Completed high school	0.75 (0.36 to 1.56)
Some post-secondary (college or	1.74 (0.85 to 3.58)
University degree	2.59 (1.16 to 5.78)
Marital Status	
Married/Living with partner	1.00 (Referent)
Previously married	0.84 (0.45 to 1.56)
Never married	1.33 (0.71 to 2.46)
Smoking Status	
Current	1.00 (Referent)
Former	1.04 (0.51 to 2.09)
Never	0.55 (0.31 to 0.99)
Religious Service Attendance, past 12 months	
None	1.00 (Referent)
1 to 6 times	0.62 (0.36 to 1.06)
7 or more times	0.88 (0.52 to 1.49)
Employment status	
Employed	1.00 (Referent)
Unemployed	1.31 (0.45 to 3.80)
Other (student, retired, homemaker)	1.18 (0.68 to 2.05)
Alcohol use in the past 12 months	0.84 (0.50 to 1.40)
Cannabis use in the past 12 months	1.25 (0.57 to 2.73)
Fair or Poor Self-reported Health	1.24 (0.62 to 2.51)
Fair or Poor Self-reported Mental Health	0.44 (0.17 to 1.10)
Health care worker	2.55 (1.08 to 6.01)
Immigrant to Canada	0.47 (0.28 to 0.81)
Urban residence	0.89 (0.56 to 1.44)

Odds ratios estimated using logistic regression for complex survey designs. Statistical significance assessed using t-distribution with 610 degrees of freedom

Participants had varied opinions about implementation of SSFs and SIFs. When asked if SSFs should be made available to people who smoke drugs such as crack cocaine and crystal methamphetamine, 64.2 % of Ontarians held a mixed overall opinion, 19.6 % strongly agreed, and 16.1 % strongly disagreed (Table 3). The distribution of responses was significantly different when participants were asked about SIFs; 28.3 % of participants strongly agreed and 11.6 % strongly disagreed. Analyses by each program goal showed significantly fewer participants strongly agreed with implementation of SSFs than with implementation of SIFs (Table 3). For example, 20.5 % strongly agreed with implementation of SSFs to encourage safer drug use compared to 30.6 % who strongly agreed with implementation of SIFs for that purpose. For each goal, significantly more participants strongly disagreed with implementation of SSFs than strongly disagreed with implementation of SIFs.

**Table 3:** Difference in opinions about SSFs and SIFS by composite measure and specific goals

	Supervised smoking	Supervised injection	Difference	*p*-value	Design Degrees of Freedom
Goal of facility^a^	Weighted percent (95 % Confidence Interval)	Weighted percent (95 % Confidence Interval)	Weighted percent (95 % Confidence Interval)		
Composite measure					
Strongly agree	19.6 % (16.5 % to 23.2 %)	28.3 % (24.6 % to 32.3 %)	-8.7 % (-12.2 % to -5.2 %)	<0.001	772
Somewhat agree/disagree	64.2 % (60.1 % to 68.2 %)	60.1 % (55.9 % to 64.2 %)	4.1 % (0.1 % to 8.1 %)	0.046	
Strongly disagree	16.1 % (13.3 % to 19.4 %)	11.6 % (9.1 % to 14.6 %)	4.6 % (2.6 % to 6.5 %)	<0.001	
Supervised smoking facilities should be made available to people who smoke drugs like crack cocaine and methamphetamine to encourage safer drug use					
Strongly agree	20.5 % (17.3 % to 24.%)	30.6 % (27.% to 34.5 %)	-10.1 % (-13.7 % to -6.6 %)	<0.001	838
Somewhat agree/disagree	40.5 % (36.5 % to 44.6 %)	40.3 % (36.3 % to 44.4 %)	0.2 % (-4.0 % to 4.3 %)	0.932	
Strongly disagree	39.1 % (35.2 % to 43.1 %)	29.1 % (25.6 % to 32.9 %)	10.% (6.9 % to 13.%)	<0.001	
Supervised smoking facilities should be made available if it can be shown that they reduce infectious disease among people who smoke drugs like crack cocaine and methamphetamine					
Strongly agree	35.2 % (31.4 % to 39.2 %)	49.4 % (45.4 % to 53.4 %)	-14.2 % (-17.7 % to -10.7 %)	<0.001	881
Somewhat agree/disagree	39.9 % (36.0 % to 43.9 %)	32.4 % (28.9 % to 36.2 %)	7.4 % (3.5 % to 11.4 %)	<0.001	
Strongly disagree	24.9 % (21.7 % to 28.4 %)	18.1 % (15.3 % to 21.4 %)	6.8 % (4.3 % to 9.3 %)	<0.001	
Supervised smoking facilities should be made available if they can increase drug users’ contact with health and social workers					
Strongly agree	40.1 % (36.3 % to 44.1 %)	48.3 % (44.4 % to 52.3 %)	-8.2 % (-11.3 % to -5.%)	<0.001	886
Somewhat agree/disagree	40.% (36.2 % to 44.%)	37.2 % (33.4 % to 41.1 %)	2.9 % (-0.5 % to 6.3 %)	0.097	
Strongly disagree	19.8 % (16.9 % to 23.1 %)	14.5 % (11.9 % to 17.5 %)	5.3 % (3.1 % to 7.5 %)	<0.001	
Supervised smoking facilities should be made available if it can be shown that they reduce neighbourhood problems related to use of drugs like crack cocaine and methamphetamine					
Strongly agree	45.7 % (41.8 % to 49.7 %)	56.1 % (52.2 % to 60.%)	-10.4 % (-13.4 % to -7.4 %)	<0.001	910
Somewhat agree/disagree	36.6 % (32.9 % to 40.4 %)	31.% (27.5 % to 34.8 %)	5.6 % (2.4 % to 8.7 %)	<0.001	
Strongly disagree	17.7 % (15.% to 20.8 %)	12.9 % (10.5 % to 15.7 %)	4.8 % (3.1 % to 6.6 %)	<0.001	

^a^Supervised injection questions were worded correspondingly to ask about drug injection. Proportions estimated using complex survey designs. Differences calculated using t-distribution for differences with specified degrees of freedom. For our survey design, sample size = design degrees of freedom + number of strata (6)

Bivariate analyses showed that just over half of Ontarians (51.1 %) somewhat agreed/disagreed, 15.7 % strongly agreed and 10.6 % strongly disagreed with implementing both SSFs and SIFs (Table 4).

**Table 4:** Bivariate analysis of composite measures of agreement regarding supervised injection and smoking facilities^a^

	Supervised smoking composite measure			
Supervised injection composite measure	Strongly agree	Somewhat agree/disagree	Strongly disagree	*p*-value
Strongly Agree	15.7 %	12.1 %	0.5 %	
Somewhat agree/disagree	3.9 %	51.1 %	5.1 %	
Strongly disagree	0.0 %	1.0 %	10.6 %	<0.001

^a^Proportions estimated using complex survey designs. Test statistic for distribution uses Pearson’s chi-square test converted to an F-statistic for the survey design with the second-order correction of Rao and Scott with F(3.90, 3011.27) = 123.7119 [46]

Prior knowledge of SSFs influenced support for implementation. Participants who had prior knowledge of SSFs were more likely to strongly agree with implementation of SSFs on the composite measure of agreement than those who did not have prior knowledge (28.4 % vs. 16.8 %; difference 11.6 % [95 % CI 2.7 % to 20.5 %]) and less likely to report mixed opinions (55.1 % vs. 66.7 %; difference -11.6 % [95 % CI -22.1 % to -1.1 %]; Table 5). Participants with prior knowledge of SSFs were also more supportive of SSF implementation in relation to the goals of encouraging safer drug use (difference 12.8 % reducing infectious diseases (difference 14.9 % and slightly more likely to support implementation for increasing contact with health and social workers (difference 11.1 %), but not significantly associated with agreement to implement SSFs to reduce neighbourhood problems (difference 6.7 % Strong disagreement with implementation of SSFs overall was not influenced by prior awareness of SSFs except for the goal of encouraging safer drug use, where participants who were not aware of SSFs were more likely to strongly disagree with implementation (41.4 % vs. 29.6 %).

**Table 5:** Agreement with goals of a supervised smoking facility by level of awareness^a^

		Aware of supervised smoking facilities				
Goal of facility	No (95 % CI)	Yes (95 % Cl)	Difference (95 % CI)	*p*-value (difference)	*p*-value (distribution)	Degrees of freedom
Composite measure						
Strongly agree	16.8 % (13.6 % to 20.6 %)	28.4 % (21.0 % to 37.3 %)	11.6 % (2.7 % to 20.5 %)	0.011		Difference: 829 Distribution: F(1.99, 1652.12) = 4.0000
Somewhat agree/disagree	66.7 % (62.3 % to 70.8 %)	55.1 % (45.4 % to 64.4 %)	-11.6 % (-22.1 % to -1.1 %)	0.030		
Strongly disagree	16.5 % (13.5 % to 20.0 %)	16.5 % (10.5 % to 25.0 %)	0.0 % (-7.9 % to 7.9 %)	0.995	0.019	
Supervised smoking facilities should be made available to people who smoke drugs like crack cocaine and methamphetamine to encourage safer drug use						
Strongly agree	17.3 % (14.2 % to 21.0 %)	30.2 % (22.7 % to 38.9 %)	12.8 % (4.0 % to 21.6 %)	0.005		Difference: 889 Distribution: F(1.97, 1752.21) = 5.3906
Somewhat agree/disagree	41.2 % (37.0 % to 45.6 %)	40.3 % (31.0 % to 50.3 %)	-0.9 % (-11.6 % to 9.8 %)	0.863		
Strongly disagree	41.4 % (37.2 % to 45.7 %)	29.6 % (22.0 % to 38.5 %)	-11.9 % (-21.2 % to -2.5 %)	0.013	0.005	
Supervised smoking facilities should be made available if it can be shown that they reduce infectious disease among people who smoke drugs like crack cocaine and methamphetamine						
Strongly agree	31.8 % (27.8 % to 36.0 %)	46.7 % (37.4 % to 56.3 %)	14.9 % (4.5 % to 25.3 %)	0.005		Difference: 910 Distribution: F(1.98, 1802.95) = 4.4720
Somewhat agree/disagree	42.3 % (38.1 % to 46.6 %)	30.5 % (22.0 % to 40.5 %)	-11.8 % (-22.1 % to -1.6 %)	0.023		
Strongly disagree	25.9 % (22.4 % to 29.7 %)	22.8 % (16.0 % to 31.4 %)	-3.1 % (-11.6 % to 5.4 %)	0.478	0.012	
Supervised smoking facilities should be made available if they can increase drug users’ contact with health and social workers						
Strongly agree	37.7 % (33.6 % to 42.0 %)	48.8 % (39.3 % to 58.4 %)	11.1 % (0.6 % to 21.6 %)	0.039		Difference: 919 Distribution: F(1.98, 1823.00) = 2.2802
Somewhat agree/disagree	42.1 % (37.9 % to 46.3 %)	33.1 % (24.3 % to 43.3 %)	-9.0 % (-19.5 % to 1.5 %)	0.093		
Strongly disagree	20.2 % (17.1 % to 23.8 %)	18.1 % (12.0 % to 26.4 %)	-2.1 % (-10.0 % to 5.8 %)	0.599	0.103	
Supervised smoking facilities should be made available if it can be shown that they reduce neighbourhood problems related to use of drugs like crack cocaine and methamphetamine						
Strongly agree	44.0 % (39.8 % to 48.2 %)	50.7 % (41.1 % to 60.2 %)	6.7 % (-3.8 % to 17.2 %)	0.213		Difference: 934 Distribution: F(1.97, 1844.31) = 1.1145
Somewhat agree/disagree	38.3 % (34.3 % to 42.4 %)	30.7 % (22.0 % to 40.9 %)	-7.6 % (-18.0 % to 2.8 %)	0.151		
Strongly disagree	17.8 % (14.9 % to 21.1 %)	18.7 % (12.5 % to 26.9 %)	0.9 % (-6.9 % to 8.8 %)	0.821	0.328	

^a^CI denotes confidence interval. Proportions estimated using complex survey designs. Differences calculated using t-distribution for differences with specified degrees of freedom. Test statistic for distribution uses Pearson’s chi-square test converted to an F-statistic for the survey design with the second-order correction of Rao and Scott with specified degrees of freedom [46]

Few variables were predictive of either strongly agreeing or strongly disagreeing with the goals of SSFs (Table 6). People with an annual income between $50,000 and $80,000 and those who attended religious services 7 or more times in the year prior to being surveyed were significantly less likely to agree with the goals of SSFs compared to those who somewhat agreed/disagreed, whereas people who smoked cannabis in the past 12 months were significantly less likely to disagree with the goals of SSFs compared to those who somewhat agreed/disagreed. Post-estimation adjustment predicted for strongly disagreeing with the goals of SIFs for people with no, 1 to 6, and 7 or more religious service attendances per year were 9.3 %, 9.5 %, and 13.1 %.

**Table 6:** Multivariable analysis of agreement with the goals of supervised smoking facilities

	Relative risk ratio (95 % Confidence Interval)	
Variable	Strongly agree vs. somewhat agree/disagree	Strongly disagree vs. somewhat agree/disagree
Age (per decade)	1.09 (0.93 to 1.27)	0.93 (0.71 to 1.23)
Male sex	1.18 (0.71 to 1.99)	1.36 (0.64 to 2.89)
Household income in the past year before taxes		
Less than $30,000	1.00 (Referent)	1.00 (Referent)
Between $30,000 and $49,999,99	0.73 (0.34 to 1.58)	0.91 (0.29 to 2.86)
Between $50,000 and $79,999,99	0.35 (0.15 to 0.81)	0.51 (0.17 to 1.52)
More than $80,000	0.85 (0.38 to 1.93)	1.32 (0.36 to 4.82)
Highest level of education attained		
Less than high school	1.00 (Referent)	1.00 (Referent)
Completed high school	1.14 (0.46 to 2.82)	0.64 (0.24 to 1.71)
Some post-secondary (college or	0.74 (0.31 to 1.81)	0.46 (0.18 to 1.22)
University degree	1.52 (0.58 to 3.98)	0.43 (0.13 to 1.40)
Marital Status		
Married/Living with partner	1.00 (Referent)	1.00 (Referent)
Previously married	0.80 (0.37 to 1.72)	1.10 (0.43 to 2.80)
Never married	1.86 (0.91 to 3.77)	1.61 (0.57 to 4.55)
Smoking Status		
Current	1.00 (Referent)	1.00 (Referent)
Former	0.79 (0.37 to 1.67)	1.04 (0.36 to 2.99)
Never	0.90 (0.48 to 1.71)	0.78 (0.31 to 1.99)
Religious Service Attendance, past 12 months		
None	1.00 (Referent)	1.00 (Referent)
1 to 6 times	0.61 (0.34 to 1.08)	0.85 (0.30 to 2.36)
7 or more times	0.24 (0.13 to 0.45)	0.89 (0.37 to 2.16)
Employment status		
Employed	1.00 (Referent)	1.00 (Referent)
Unemployed	0.86 (0.28 to 2.67)	0.86 (0.14 to 5.06)
Other (student, retired, homemaker)	0.97 (0.53 to 1.76)	0.69 (0.26 to 1.81)
Alcohol use in the past 12 months	0.71 (0.36 to 1.37)	0.78 (0.35 to 1.75)
Cannabis use in the past 12 months	0.81 (0.38 to 1.72)	0.16 (0.03 to 0.76)
Fair or Poor Self-reported Health	0.96 (0.39 to 2.37)	1.03 (0.30 to 3.50)
Fair or Poor Self-reported Mental Health	0.68 (0.26 to 1.77)	0.66 (0.12 to 3.67)
Health care worker	1.37 (0.59 to 3.17)	0.21 (0.02 to 1.79)
Immigrant to Canada	0.83 (0.44 to 1.55)	1.49 (0.63 to 3.57)
Urban residence	1.12 (0.62 to 2.02)	0.71 (0.34 to 1.51)

Relative risk ratios estimated using multinomial logistic regression for complex survey designs. Statistical significance assessed using t-distribution with 536 degrees of freedom

## Discussion

Although there are reports of decline in crack cocaine use in both Canada and the United States [39, 40], its use remains a public health concern. However, as noted above, public health programming for people who smoke crack cocaine lags behind what is available for people who inject drugs despite evidence of serious, and sometimes fatal, harms associated with smoking these drugs [41, 42]. To our knowledge, this is the first study to report public opinion about SSFs.

Lack of innovative public health responses to reduce stimulant drug-related problems parallels the lack of awareness about SSFs in the public opinion data. Our results show that less than 20 % of Ontarians were aware of SSF models whereas almost 60 % were aware of SIF models. SSF awareness was associated with older age, higher education and male sex. These results suggest the need for education among broad societal groups regarding supervised smoking. Compared to smoking facilities, we generally found stronger support for and fewer negative sentiments about injection facilities. Support for SSFs was strongest when the goals of a facility were presented as reducing neighbourhood problems related to drug use, increasing contact between people who use drugs and health and social workers, and reducing infectious diseases. Even for these goals, however, we observed about 8 to 14 % higher support for injection facilities, rather than smoking facilities. We found few consistent predictors of opposition to SSF goals although regular attendance at religious services was significant. These findings might indicate that both individual values and scepticism about the effectiveness of SSFs, reflecting the weaker evidence base for SSFs compared to SIFs, underlie opposition. Our results underscore the need for ongoing research, including demonstration projects.

The survey was prone to sampling biases, as people who do not speak English or French and residents without a landline were excluded from participating. Nevertheless, the response rate was reasonable (57 %) for a telephone survey [35, 43] and the sampling was systematic. Thus, these data are population-based – unlike other studies assessing public opinion about SIFs that use convenience samples – and therefore more likely to be representative of true opinion, although highly educated people were somewhat over-represented, as commonly found in telephone surveys. The survey represents the last available data (in 2009), but SIFs have remained a prominent policy discussion topic since then. While our results might underestimate awareness of SIFs, they likely are still reflective of awareness of SSFs, which have been proposed but not implemented in Canada and are discussed much less frequently.

Grund and colleagues [22] note that stimulant use presents unique challenges requiring innovative and multidisciplinary responses and that people who use stimulants ‘must enjoy the fundamental human right to health protection, as stipulated by Article 25 of the Universal Declaration of Human Rights’ (p. 212). SSFs represent an innovation in programming that have been implemented in some European cities [9]. Until recently, harm reduction advocates in Canada have despaired over introducing innovations such as these given the opposition of the former Conservative federal government to harm reduction [44]. Again, our data also show low levels of public support for SSFs. Importantly, public opinion is identified in recent legislation, Bill C-2, passed in Canada that identified stakeholder opinion as one of the inputs that would be considered in any application for new SIFs [http://www.huffingtonpost.ca/2015/06/22/critics-up-in-arms-over-f_n_7640154.html]. However, public health advocates are hopeful that another SIF recently approved by the new federal government signals improved chances for approval of other applications for SIFs. [http://www.theglobeandmail.com/news/british-columbia/vancouver-facility-becomes-canadas-second-approved-supervised-injection-site/article28216557/]. The newly elected Prime Minister Justin Trudeau is on record expressing support for SIFs [http://www.straight.com/blogra/404631/justin-trudeau-tells-ubc-students-he-wants-supervised-injection-sites-across-canada], reflecting the leadership identified as necessary for innovation in programming and policy [45].

## Conclusions

The lack of public knowledge or support of SSFs and their potential positive impact on public health issues associated with drug smoking presents barriers to service providers and community advocates who are contemplating implementation of SSFs in their jurisdiction. Recent federal government changes in Canada may provide the leadership environment necessary to ensure that innovative harm reduction programs such as SSFs are developed based on evidence and implemented.

## Abbreviations

SIF: Supervised injection facility: a common type of supervised consumption facility where people can inject illicitly obtained drugs
SSF: Supervised smoking facility: type of supervised consumption facility where people can smoke illicitly obtained drugs
